# Correspondence to the *EJPC* in response to position paper by Ambrosetti M et al. 2020: Cardiovascular rehabilitation and COVID-19: The need to maintain access to evidence-based services from the safety of home

**DOI:** 10.1177/2047487320923053

**Published:** 2020-04-30

**Authors:** Hasnain Dalal, Rod S Taylor, Colin Greaves, Patrick J Doherty, Sinead TJ McDonagh, Samantha B van Beurden, Carrie Purcell

**Affiliations:** Royal Cornwall Hospitals NHS Trust, Truro, UK; College of Medicine and Health, University of Exeter, UK; MRC/CSO Social and Public Health Sciences Unit & Robertson Centre for Biostatistics, Institute of Health and Well Being, University of Glasgow, UK; School of Sport, Exercise & Rehabilitation Sciences, University of Birmingham, UK; Department of Health Sciences, University of York, UK

Sir,

The 2020 update position paper on secondary prevention and cardiovascular rehabilitation from the Secondary Prevention and Rehabilitation Section of the European Association of Preventive Cardiology is welcome. However, the paper is wanting for the lack of a firm recommendation for home-based cardiac rehabilitation especially as these programmes are highly relevant in the era of coronavirus disease 2019–COVID-19.^1^

Traditionally, the majority of patients receive cardiac rehabilitation in supervised, group-based classes conducted in hospital outpatient departments or community centres, ranging in frequency, intensity and duration of exercise and self-help guidance over several weeks. Participation rates in centre-based cardiac rehabilitation programmes remain suboptimal, despite current national and international recommendations.^2^ Over the last decade, there have been calls for new strategies to improve participation in cardiac rehabilitation; home- or web-based options have been suggested, several have been trialled and tested,^2,3^ and some of these approaches have been reiterated in the position paper.^1^ The American College of Cardiology/American Heart Association recently endorsed home-based rehabilitation,^4^ and the updated UK National Institute for Health and Care Excellence 2018 guidance on chronic heart failure also stated that ‘delivery of home-based rehabilitation may increase access and uptake’.^5^ The flexibility and better adherence of engaging in home-based cardiac rehabilitation are given as reasons for potential benefit.^4^ There is a surprise omission from the position paper,^1^ however, of a UK multicentre trial of home-based rehabilitation in heart failure. The findings of the Rehabilitation Enablement in Chronic Heart Failure (REACH-HF) study have demonstrated clinical effectiveness and cost effectiveness and have been published in this journal.^6,7^ The British Heart Foundation has just awarded a grant to co-develop a digital version of REACH HF to improve access to rehabilitation to patients with heart failure in the UK.

The impact of the COVID-19 pandemic on health and healthcare systems in most European countries and North America has meant that the general public have been advised to abide by social distancing guidance. This can be achieved by staying at home, to prevent the spread of the virus. Patients with heart disease are at high risk of severe complications from COVID-19 infection, and, therefore, have been advised to self-isolate to lower the risk of contracting the virus. For these individuals, the ability to exercise and follow a self-care rehabilitation intervention without leaving the home has never been more pertinent.

Home-based cardiac rehabilitation programmes can also be delivered digitally and remotely.^8^ They thus reduce need for face-to-face contact, and provide an easily accessible platform for healthcare professionals to facilitate delivery of rehabilitation, including interaction with patients and caregivers, without the need for travel. The NHS Lothian Heart Manual, a home-based cardiac rehabilitation programme dating back to the 1990s, is ‘perhaps the most extensively studied self-management book for patients recovering from acute myocardial infarction or coronary revascularization’^4^ and is now available in digital format (www.theheartmanual.com).

The COVID-19 crisis is moving us in a direction where self-managed, home-based interventions are being encouraged, and are likely to remain with us beyond the current pandemic. Research on the implementation of novel cardiac rehabilitation approaches is urgently needed to help guide changes in future cardiac rehabilitation services and maintain provision of patient care.

In response to the outbreak of COVID-19, the American Association of Cardiovascular and Pulmonary Rehabilitation has established the Innovative Delivery Model Collaborative to facilitate digital approaches and encourage home-based delivery of cardiac rehabilitation, supported by several providers, including the Mayo Clinic (http://www.aacvpr.org/covid19). However, caution is required when introducing innovative modes of delivery, and consideration should be given both to ‘patient-related barriers for digital health deployment’^1^ and to the lack of robust evaluation of the new modes of delivery.

The urgency of maintaining access to evidence-based cardiac rehabilitation services from the safety of home, particularly during the current global pandemic, could not be greater. We need to act now.^9^
